# Beyond universality: confronting nursing theories with the realities of chronic care

**DOI:** 10.1590/1518-8345.0000.4994

**Published:** 2026-04-27

**Authors:** Omar Pereira de Almeida, Ercole Vellone, Barbara Riegel, Eneida Rejane Rabelo-Silva, Valentina Zeffiro, Valerio Della-Bella

**Affiliations:** 1 Federal University of Uberlandia, School of Medicine, Uberlandia, MG, Brazil.; 2 Tor Vergata University of Rome, Department of Biomedicine and Prevention, Rome, RM, Italy.; 3 Wroclaw Medical University, Faculty of Health Sciences, Wroclaw, DS, Poland.; 4 University of Pennsylvania, School of Nursing, Philadelphia, PA, United States of America.; 5 Federal University of Rio Grande do Sul, School of Nursing, Porto Alegre, RS, Brazil.; 6 University Hospital of Rome Tor Vergata , Nursing Department, Rome, RM, Italy.

**Figure:**
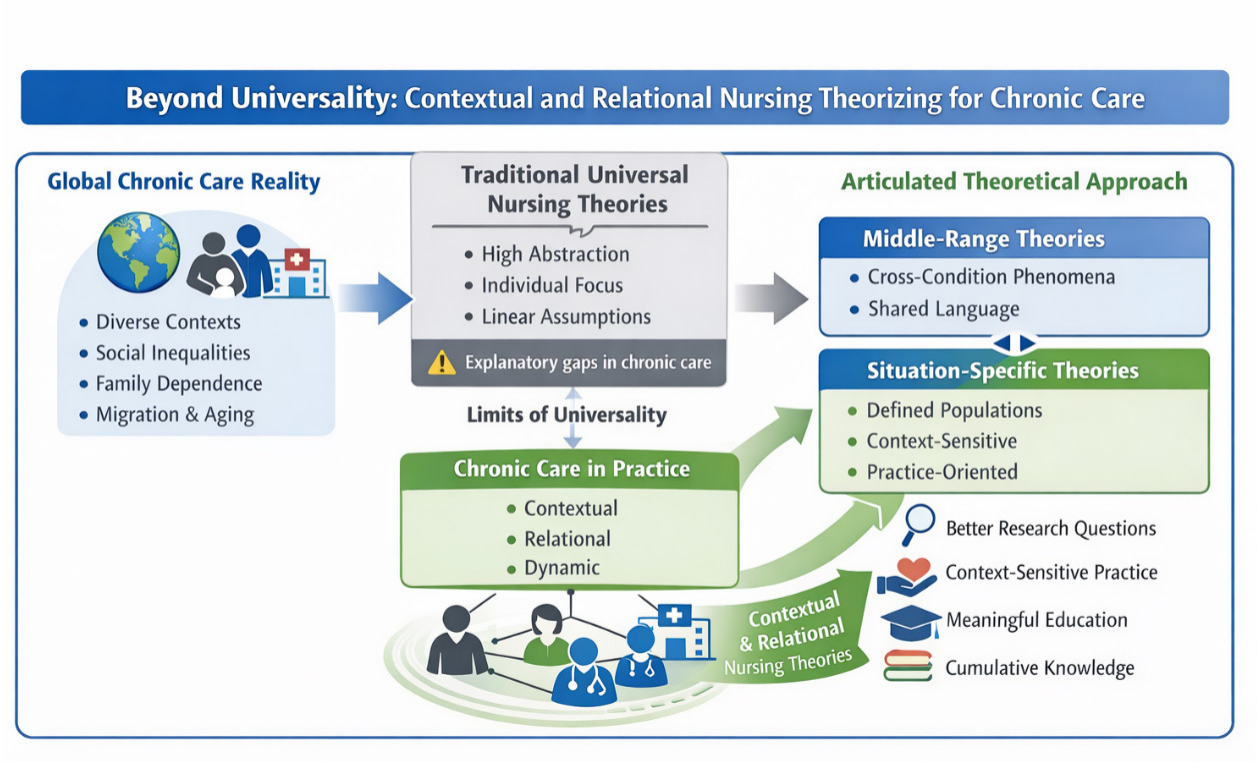

**Figure:**
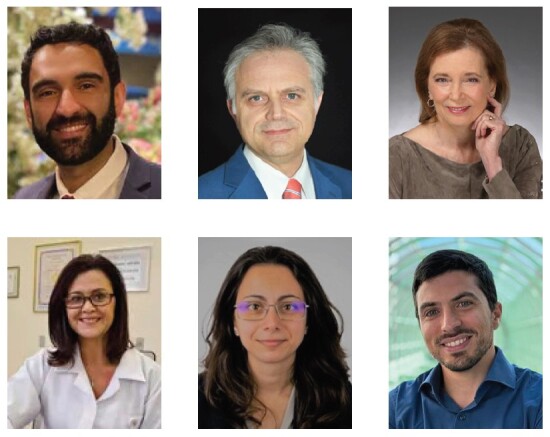

Chronic conditions have become a significant problem for health systems around the globe. No matter the location, nurses are increasingly in charge of caring for individuals, families, and communities dealing with ongoing health issues^1^. While medical diagnoses may be alike in different countries, the experience of living with chronic illness varies widely. Social inequalities, family dynamics, health system structure, cultural practices, and available resources greatly influence how chronic care is experienced in daily life^1^ ^-^ ^2^.

This growing dissonance between global clinical characteristics and local care realities raises a fundamental question for nursing science: how are nursing theories understood and applied to adequately explain and guide chronic care as it is actually delivered in practice?

## The limits of universalization in chronic care

For decades, nursing theories have aspired to universality^2^. Grand theories and, subsequently, middle-range theories have sought to identify concepts and relationships applicable across different contexts, populations, and cultures^2^ ^-^ ^3^. Middle-range theories represent a significant advance by capturing phenomena that cut across chronic conditions, although they may benefit from further developments that incorporate specific clinical and relational contexts^2^.

In contrast, universal and very abstract theories usually work best in situations with relative stability and clearly defined phenomena, conditions that are seldom found in chronic care^1^ ^-^ ^2^. The difference between theoretical ideas and the realities of care becomes especially clear in settings where chronic conditions are influenced by social inequalities and a heavy reliance on informal caregiving.

In South America, for instance, chronic care frequently occurs in environments marked by structural disadvantages including unequal access to healthcare services, socioeconomic and territorial inequalities, and a strong dependence on family networks^4^ ^-^ ^5^. In high-income countries, population aging and migration generate different, yet equally complex, challenges^2^. Despite these diverse realities, theoretical models often assume stable environments, autonomous individuals, and linear care processes^3^.

This tension reveals an epistemological challenge. When theories strive for universal applicability, they may sacrifice sensitivity to context and relational dynamics. In chronic care, this frequently results in explanatory gaps: why interventions succeed in one setting and fail in another, why individuals with similar diagnoses experience divergent outcomes, or why “evidence-based” recommendations are inconsistently adopted in practice.

## Chronic care as a fundamentally relational phenomenon

Beyond the boundaries of universalization, chronic care reveals another ongoing blind spot in nursing theory: individualism. Many theoretical frameworks still focus mainly on the individual patient as the main unit of analysis^1^ ^-^ ^2^. Concepts such as self-care, treatment adherence, and symptom management are often operationalized as individual capacities or responsibilities^3^ ^,^ ^5^. However, chronic care is rarely an individual endeavor.

In daily practice, managing chronic illness happens through relationships. Family members and informal caregivers watch symptoms, discuss treatment choices, offer emotional support, and help with functional limitations^2^ ^-^ ^3^. Informal caregivers often fill voids left by health systems, especially in situations with limited resources^4^. Consequently, chronic care is influenced by continuous interactions among patients, caregivers, health professionals, and institutions^2^ ^-^ ^5^.

Chronic care therefore challenges nursing theory to become not only more contextual, but also more explicitly relational.

## Middle-range and situation-specific theories as complementary responses

Addressing the complexity of chronic care does not require abandoning theory, but rather aligning levels of theoretical abstraction with the nature of the phenomenon under study^2^. In this regard, middle-range and situation-specific nursing theories offer complementary, practice-oriented responses to the limitations of grand theories^3^ ^,^ ^5^.

Middle-range theories are particularly well suited to phenomena that cut across multiple chronic conditions, such as adaptation, self-care, informal caregiving, and illness trajectories^2^. They provide a shared conceptual language that enables comparison and accumulation of knowledge across different diagnoses, while maintaining a level of abstraction that remains meaningful for practice^1^ ^-^ ^2^.

Situation-specific nursing theories are particularly effective in chronic conditions or specific care situations because they are grounded in defined populations, contexts, and relational configurations, allowing them to explain how clinical, social, familial, cultural, and health system factors interact in care processes, while providing conceptually rigorous insights directly linked to clinical decision-making and intervention design^2^ ^,^ ^5^.

In chronic care, these two theoretical levels should not be viewed as competing, but as mutually reinforcing. Middle-range theories provide coherence across the broad landscape of chronicity, while situation-specific theories translate this coherence into models that are sensitive to disease-specific trajectories and the concrete realities of care^2^.

From a transnational perspective, situation-specific theories promote dialogue rather than fragment nursing knowledge by linking different contexts through shared caregiving experiences, enabling comparison, refinement, and cumulative theory building grounded in real-world complexity.

## Implications for nursing science, practice, and education

Adopting an articulated use of middle-range and situation-specific theories in chronic care has important implications^1^ ^-^ ^2^. For research, this approach encourages study designs that prioritize contextual variables, relational outcomes, and longitudinal processes. Rather than asking whether an intervention “works” in general, nursing science can more meaningfully ask for whom, under what conditions, and through which relationships it works, for example through the use of targeted statistical approaches.

For practice, situation-specific theories provide conceptual tools that are immediately interpretable by clinical nurses^1^ ^-^ ^2^ ^,^ ^5^. They support interventions that are adaptable rather than prescriptive, sensitive to family dynamics, and responsive to social constraints^5^.

For education, teaching nursing theory through middle-range and situation-specific frameworks helps students understand theory as a living, practice-oriented resource rather than an abstract requirement^2^. This positions nurses not only as consumers of theory, but also as legitimate producers of contextually grounded knowledge^1^.

## A call for coherent, situated, and relational theorizing in chronic care

The global burden of chronic conditions demands theoretical approaches capable of capturing complexity without sacrificing coherence^1^. Nursing theory for chronic care must therefore move beyond the pursuit of universality and advance toward conceptual coherence across different levels of abstraction^2^.

By articulating middle-range and situation-specific theories, nursing science can more accurately reflect how chronic care is actually lived, negotiated, and sustained^3^ ^,^ ^5^. The challenge of contemporary theoretical production in nursing is not to create theories that erase differences, but theories that learn from them.

In chronic care, nursing theories cannot be preserved merely as intellectual heritage; they must be continuously confronted with the clinical and social foundations of care, or risk becoming conceptually elegant yet scientifically irrelevant.
