# Supplementary material for: Beyond universality: confronting nursing theories with the realities of chronic care
Source: Rev Lat Am Enfermagem. 2026 Apr 27;34:e4994. doi: 10.1590/1518-8345.0000.4994 (PMC13124037; doi:10.1590/1518-8345.0000.4994)
Supplement: Translation

# Translation: Além da universalidade: confrontando as teorias de enfermagem com as realidades do cuidado às condições crônicas

**DOI:** 10.1590/1518-8345.0000.4995

**Authors:** Omar Pereira de Almeida, Ercole Vellone, Barbara Riegel, Eneida Rejane Rabelo-Silva, Valentina Zeffiro, Valerio Della-Bella

**Affiliations:** 1 Federal University of Uberlandia, School of Medicine, Uberlandia, MG, Brazil.; 2 Tor Vergata University of Rome, Department of Biomedicine and Prevention, Rome, RM, Italy.; 3 Wroclaw Medical University, Faculty of Health Sciences, Wroclaw, DS, Poland.; 4 University of Pennsylvania, School of Nursing, Philadelphia, PA, United States of America.; 5 Federal University of Rio Grande do Sul, School of Nursing, Porto Alegre, RS, Brazil.; 6 University Hospital of Rome Tor Vergata , Nursing Department, Rome, RM, Italy.

As condições crônicas tornaram-se um problema significativo para os sistemas de saúde em todo o mundo. Independentemente da localização, os enfermeiros estão cada vez mais responsáveis pelo cuidado de indivíduos, famílias e comunidades que lidam com problemas de saúde contínuos^1^. Embora os diagnósticos médicos possam ser semelhantes em diferentes países, a experiência de viver com uma doença crônica varia amplamente. Desigualdades sociais, dinâmicas familiares, estrutura dos sistemas de saúde, práticas culturais e recursos disponíveis influenciam profundamente a forma como o cuidado às condições crônicas é vivenciado na vida cotidiana^1^ ^-^ ^2^.

Essa crescente dissonância entre as características clínicas globais e as realidades locais do cuidado levanta uma questão fundamental para a ciência da enfermagem: como as teorias de enfermagem são compreendidas e aplicadas para explicar e orientar adequadamente o cuidado às condições crônicas tal como ele é efetivamente realizado na prática?

## Os limites da universalização no cuidado às condições crônicas

Por décadas, as teorias de enfermagem aspiraram à universalidade^2^. As grandes teorias e, posteriormente, as teorias de médio alcance buscaram identificar conceitos e relações aplicáveis a diferentes contextos, populações e culturas^2^ ^-^ ^3^. As teorias de médio alcance representam um avanço significativo ao captar fenômenos que atravessam diferentes condições crônicas, embora possam se beneficiar de desenvolvimentos adicionais que incorporem contextos clínicos e relacionais específicos^2^.

Em contraste, teorias universais e muito abstratas tendem a funcionar melhor em situações de relativa estabilidade e com fenômenos claramente definidos, condições que raramente são encontradas no cuidado às condições crônicas^1^ ^-^ ^2^. A diferença entre as ideias teóricas e as realidades do cuidado torna-se especialmente evidente em contextos nos quais as condições crônicas são influenciadas por desigualdades sociais e por uma forte dependência do cuidado informal.

Na América do Sul, por exemplo, o cuidado às condições crônicas frequentemente ocorre em ambientes marcados por desvantagens estruturais, incluindo acesso desigual aos serviços de saúde, desigualdades socioeconômicas e territoriais, e forte dependência das redes familiares^4^ ^-^ ^5^. Em países de alta renda, o envelhecimento populacional e a migração geram desafios diferentes, mas igualmente complexos^2^. Apesar dessas realidades diversas, os modelos teóricos frequentemente pressupõem ambientes estáveis, indivíduos autônomos e processos de cuidado lineares^3^.

Essa tensão revela um desafio epistemológico. Quando as teorias buscam aplicabilidade universal, podem sacrificar a sensibilidade ao contexto e às dinâmicas relacionais. No cuidado às condições crônicas, isso frequentemente resulta em lacunas explicativas: por que intervenções têm sucesso em um contexto e falham em outro, por que indivíduos com diagnósticos semelhantes apresentam desfechos divergentes, ou por que recomendações “baseadas em evidências” são adotadas de forma inconsistente na prática.

## O cuidado às condições crônicas como um fenômeno fundamentalmente relacional

Para além dos limites da universalização, o cuidado às condições crônicas revela outro ponto cego persistente na teoria de enfermagem: o individualismo. Muitos referenciais teóricos ainda se concentram principalmente no paciente individual como principal unidade de análise^1^ ^-^ ^2^. Conceitos como autocuidado, adesão ao tratamento e manejo de sintomas são frequentemente operacionalizados como capacidades ou responsabilidades individuais^3^ ^,^ ^5^. Entretanto, o cuidado às condições crônicas raramente é um empreendimento individual.

Na prática cotidiana, o manejo da doença crônica ocorre por meio de relações. Familiares e cuidadores informais observam sintomas, discutem escolhas terapêuticas, oferecem apoio emocional e ajudam a lidar com limitações funcionais^2^ ^-^ ^3^. Cuidadores informais frequentemente preenchem lacunas deixadas pelos sistemas de saúde, especialmente em contextos de recursos limitados^4^. Consequentemente, o cuidado às condições crônicas é influenciado por interações contínuas entre pacientes, cuidadores, profissionais de saúde e instituições^2^ ^-^ ^5^.

O cuidado às condições crônicas desafia, portanto, a teoria de enfermagem a tornar-se não apenas mais contextual, mas também mais explicitamente relacional.

## Teorias de médio alcance e teorias específicas de situação como respostas complementares

Enfrentar a complexidade do cuidado às condições crônicas não exige abandonar a teoria, mas sim alinhar os níveis de abstração teórica à natureza do fenômeno estudado^2^. Nesse sentido, as teorias de médio alcance e as teorias de enfermagem específicas de situação oferecem respostas complementares e orientadas para a prática às limitações das grandes teorias^3^ ^,^ ^5^.

As teorias de médio alcance são particularmente adequadas para fenômenos que atravessam múltiplas condições crônicas, como adaptação, autocuidado, cuidado informal e trajetórias de doença^2^. Elas fornecem uma linguagem conceitual compartilhada que possibilita comparação e acúmulo de conhecimento entre diferentes diagnósticos, mantendo ao mesmo tempo um nível de abstração que permanece significativo para a prática^1^ ^-^ ^2^.

As teorias de enfermagem específicas de situação são especialmente eficazes em condições crônicas ou situações de cuidado específicas porque estão fundamentadas em populações, contextos e configurações relacionais definidos. Isso lhes permite explicar como fatores clínicos, sociais, familiares, culturais e relacionados ao sistema de saúde interagem nos processos de cuidado, ao mesmo tempo em que oferecem *insights* conceitualmente rigorosos diretamente vinculados à tomada de decisão clínica e ao desenho de intervenções^2^ ^,^ ^5^.

No cuidado às condições crônicas, esses dois níveis teóricos não devem ser vistos como concorrentes, mas como mutuamente reforçadores. As teorias de médio alcance oferecem coerência ao amplo panorama da cronicidade, enquanto as teorias específicas de situação traduzem essa coerência em modelos sensíveis às trajetórias específicas das doenças e às realidades concretas do cuidado^2^.

De uma perspectiva transnacional, as teorias específicas de situação promovem diálogo, em vez de fragmentar o conhecimento em enfermagem, ao conectar diferentes contextos por meio de experiências compartilhadas de cuidado, permitindo comparação, refinamento e construção cumulativa de teorias fundamentadas na complexidade do mundo real.

## Implicações para a ciência, a prática e a educação em enfermagem

Adotar um uso articulado de teorias de médio alcance e teorias específicas de situação no cuidado às condições crônicas traz importantes implicações^1^ ^-^ ^2^. Para a pesquisa, essa abordagem incentiva desenhos de estudo que priorizem variáveis contextuais, desfechos relacionais e processos longitudinais. Em vez de perguntar se uma intervenção “funciona” de forma geral, a ciência da enfermagem pode perguntar de maneira mais significativa para quem, sob quais condições e por meio de quais relações ela funciona, por exemplo, por meio do uso de abordagens estatísticas direcionadas.

Para a prática, as teorias específicas de situação fornecem ferramentas conceituais imediatamente interpretáveis pelos enfermeiros clínicos^1^ ^-^ ^2^ ^,^ ^5^. Elas apoiam intervenções que são adaptáveis, em vez de prescritivas, sensíveis às dinâmicas familiares e responsivas às restrições sociais^5^.

Para a educação, ensinar teoria de enfermagem por meio de referenciais de médio alcance e específicos de situação ajuda os estudantes a compreender a teoria como um recurso vivo e orientado para a prática, e não apenas como um requisito abstrato^2^. Isso posiciona os enfermeiros não apenas como consumidores de teoria, mas também como produtores legítimos de conhecimento contextualizado^1^.

## Um chamado para uma teorização coerente, situada e relacional no cuidado às condições crônicas

A carga global das condições crônicas exige abordagens teóricas capazes de capturar a complexidade sem sacrificar a coerência^1^. A teoria de enfermagem voltada ao cuidado às condições crônicas deve, portanto, ir além da busca pela universalidade e avançar em direção à coerência conceitual entre diferentes níveis de abstração^2^.

Ao articular teorias de médio alcance e teorias específicas de situação, a ciência da enfermagem pode refletir com maior precisão como o cuidado às condições crônicas é realmente vivido, negociado e sustentado^3^ ^,^ ^5^. O desafio da produção teórica contemporânea em enfermagem não é criar teorias que apaguem as diferenças, mas teorias que aprendam com elas.

No cuidado às condições crônicas, as teorias de enfermagem não podem ser preservadas apenas como herança intelectual; elas precisam ser continuamente confrontadas com os fundamentos clínicos e sociais do cuidado, sob risco de se tornarem conceitualmente elegantes, porém cientificamente irrelevantes.

# Translation: Más allá de la universalidad: confrontando las teorías de enfermería con las realidades del cuidado de las condiciones crónicas

**DOI:** 10.1590/1518-8345.0000.4993

**Authors:** Omar Pereira de Almeida, Ercole Vellone, Barbara Riegel, Eneida Rejane Rabelo-Silva, Valentina Zeffiro, Valerio Della-Bella

**Affiliations:** 1 Federal University of Uberlandia, School of Medicine, Uberlandia, MG, Brazil.; 2 Tor Vergata University of Rome, Department of Biomedicine and Prevention, Rome, RM, Italy.; 3 Wroclaw Medical University, Faculty of Health Sciences, Wroclaw, DS, Poland.; 4 University of Pennsylvania, School of Nursing, Philadelphia, PA, United States of America.; 5 Federal University of Rio Grande do Sul, School of Nursing, Porto Alegre, RS, Brazil.; 6 University Hospital of Rome Tor Vergata , Nursing Department, Rome, RM, Italy.

Las condiciones crónicas se han convertido en un problema significativo para los sistemas de salud en todo el mundo. Independientemente del lugar, las enfermeras y los enfermeros están cada vez más a cargo del cuidado de individuos, familias y comunidades que enfrentan problemas de salud persistentes^1^. Aunque los diagnósticos médicos pueden ser similares en diferentes países, la experiencia de vivir con una enfermedad crónica varía ampliamente. Las desigualdades sociales, las dinámicas familiares, la estructura de los sistemas de salud, las prácticas culturales y los recursos disponibles influyen profundamente en la manera en que el cuidado de las condiciones crónicas se experimenta en la vida cotidiana^1^ ^-^ ^2^.

Esta creciente disonancia entre las características clínicas globales y las realidades locales del cuidado plantea una pregunta fundamental para la ciencia de la enfermería: ¿cómo se comprenden y aplican las teorías de enfermería para explicar y orientar adecuadamente el cuidado de las condiciones crónicas tal como se lleva a cabo en la práctica?

## Los límites de la universalización en el cuidado de las condiciones crónicas

Durante décadas, las teorías de enfermería han aspirado a la universalidad^2^. Las grandes teorías y, posteriormente, las teorías de rango medio han buscado identificar conceptos y relaciones aplicables a diferentes contextos, poblaciones y culturas^2^ ^-^ ^3^. Las teorías de rango medio representan un avance significativo al captar fenómenos que atraviesan distintas condiciones crónicas, aunque pueden beneficiarse de desarrollos adicionales que incorporen contextos clínicos y relacionales específicos^2^.

En contraste, las teorías universales y muy abstractas suelen funcionar mejor en situaciones de relativa estabilidad y con fenómenos claramente definidos, condiciones que rara vez se encuentran en el cuidado de las condiciones crónicas^1^ ^-^ ^2^. La diferencia entre las ideas teóricas y las realidades del cuidado se vuelve especialmente evidente en contextos donde las condiciones crónicas están influenciadas por desigualdades sociales y por una fuerte dependencia del cuidado informal.

En América del Sur, por ejemplo, el cuidado de las condiciones crónicas ocurre con frecuencia en entornos marcados por desventajas estructurales, incluyendo acceso desigual a los servicios de salud, desigualdades socioeconómicas y territoriales, y una fuerte dependencia de las redes familiares^4^ ^-^ ^5^. En los países de altos ingresos, el envejecimiento poblacional y la migración generan desafíos diferentes, aunque igualmente complejos^2^. A pesar de estas diversas realidades, los modelos teóricos a menudo suponen entornos estables, individuos autónomos y procesos de cuidado lineales^3^.

Esta tensión revela un desafío epistemológico. Cuando las teorías buscan aplicabilidad universal, pueden sacrificar la sensibilidad al contexto y a las dinámicas relacionales. En el cuidado de las condiciones crónicas, esto frecuentemente se traduce en vacíos explicativos: por qué las intervenciones tienen éxito en un contexto y fracasan en otro, por qué individuos con diagnósticos similares experimentan resultados divergentes, o por qué las recomendaciones “basadas en la evidencia” se adoptan de manera inconsistente en la práctica.

## El cuidado de las condiciones crónicas como un fenómeno fundamentalmente relacional

Más allá de los límites de la universalización, el cuidado de las condiciones crónicas revela otro punto ciego persistente en la teoría de enfermería: el individualismo. Muchos marcos teóricos todavía se centran principalmente en el paciente individual como la principal unidad de análisis^1^ ^-^ ^2^. Conceptos como el autocuidado, la adherencia al tratamiento y el manejo de los síntomas suelen operacionalizarse como capacidades o responsabilidades individuales^3^ ^,^ ^5^. Sin embargo, el cuidado de las condiciones crónicas rara vez es un esfuerzo individual.

En la práctica cotidiana, el manejo de la enfermedad crónica ocurre a través de relaciones. Los familiares y cuidadores informales observan los síntomas, discuten las decisiones terapéuticas, ofrecen apoyo emocional y ayudan a enfrentar las limitaciones funcionales^2^ ^-^ ^3^. Los cuidadores informales a menudo llenan los vacíos dejados por los sistemas de salud, especialmente en contextos con recursos limitados^4^. En consecuencia, el cuidado de las condiciones crónicas está influido por interacciones continuas entre pacientes, cuidadores, profesionales de la salud e instituciones^2^ ^-^ ^5^.

Por lo tanto, el cuidado de las condiciones crónicas desafía a la teoría de enfermería a volverse no solo más contextual, sino también más explícitamente relacional.

## Teorías de rango medio y teorías específicas de situación como respuestas complementarias

Abordar la complejidad del cuidado de las condiciones crónicas no requiere abandonar la teoría, sino alinear los niveles de abstracción teórica con la naturaleza del fenómeno estudiado^2^. En este sentido, las teorías de rango medio y las teorías de enfermería específicas de situación ofrecen respuestas complementarias y orientadas a la práctica frente a las limitaciones de las grandes teorías^3^ ^,^ ^5^.

Las teorías de rango medio son particularmente adecuadas para fenómenos que atraviesan múltiples condiciones crónicas, como la adaptación, el autocuidado, el cuidado informal y las trayectorias de la enfermedad^2^. Proporcionan un lenguaje conceptual compartido que permite la comparación y la acumulación de conocimiento entre diferentes diagnósticos, manteniendo al mismo tiempo un nivel de abstracción que sigue siendo significativo para la práctica^1^ ^-^ ^2^.

Las teorías de enfermería específicas de situación son especialmente eficaces en condiciones crónicas o situaciones de cuidado específicas porque se fundamentan en poblaciones, contextos y configuraciones relacionales definidos, lo que les permite explicar cómo los factores clínicos, sociales, familiares, culturales y del sistema de salud interactúan en los procesos de cuidado, al mismo tiempo que ofrecen aportes conceptualmente rigurosos directamente vinculados con la toma de decisiones clínicas y el diseño de intervenciones^2^ ^,^ ^5^.

En el cuidado de las condiciones crónicas, estos dos niveles teóricos no deben verse como competidores, sino como mutuamente reforzadores. Las teorías de rango medio proporcionan coherencia al amplio panorama de la cronicidad, mientras que las teorías específicas de situación traducen esa coherencia en modelos sensibles a las trayectorias específicas de las enfermedades y a las realidades concretas del cuidado^2^.

Desde una perspectiva transnacional, las teorías específicas de situación promueven el diálogo en lugar de fragmentar el conocimiento de enfermería, al vincular diferentes contextos a través de experiencias compartidas de cuidado, lo que permite la comparación, el refinamiento y la construcción acumulativa de teorías basadas en la complejidad del mundo real.

## Implicaciones para la ciencia, la práctica y la educación en enfermería

Adoptar un uso articulado de teorías de rango medio y teorías específicas de situación en el cuidado de las condiciones crónicas tiene importantes implicaciones^1^ ^-^ ^2^. Para la investigación, este enfoque fomenta diseños de estudio que prioricen variables contextuales, resultados relacionales y procesos longitudinales. En lugar de preguntar si una intervención “funciona” en general, la ciencia de la enfermería puede preguntar de manera más significativa para quién, bajo qué condiciones y a través de qué relaciones funciona, por ejemplo, mediante el uso de enfoques estadísticos específicos.

Para la práctica, las teorías específicas de situación proporcionan herramientas conceptuales que son inmediatamente interpretables por las enfermeras y los enfermeros clínicos^1^ ^-^ ^2^ ^,^ ^5^. Apoyan intervenciones adaptables en lugar de prescriptivas, sensibles a las dinámicas familiares y capaces de responder a las restricciones sociales^5^.

Para la educación, enseñar teoría de enfermería a través de marcos de rango medio y específicos de situación ayuda a los estudiantes a comprender la teoría como un recurso vivo y orientado a la práctica, y no como un requisito abstracto^2^. Esto posiciona a los profesionales de enfermería no solo como consumidores de teoría, sino también como productores legítimos de conocimiento contextualizado^1^.

## Un llamado a una teorización coherente, situada y relacional en el cuidado de las condiciones crónicas

La carga global de las condiciones crónicas exige enfoques teóricos capaces de captar la complejidad sin sacrificar la coherencia^1^. La teoría de enfermería para el cuidado de las condiciones crónicas debe, por lo tanto, ir más allá de la búsqueda de universalidad y avanzar hacia una coherencia conceptual entre diferentes niveles de abstracción^2^.

Al articular teorías de rango medio y teorías específicas de situación, la ciencia de la enfermería puede reflejar con mayor precisión cómo el cuidado de las condiciones crónicas es realmente vivido, negociado y sostenido^3^ ^,^ ^5^. El desafío de la producción teórica contemporánea en enfermería no es crear teorías que eliminen las diferencias, sino teorías que aprendan de ellas.

En el cuidado de las condiciones crónicas, las teorías de enfermería no pueden preservarse únicamente como un patrimonio intelectual; deben confrontarse continuamente con los fundamentos clínicos y sociales del cuidado, o corren el riesgo de convertirse en propuestas conceptualmente elegantes pero científicamente irrelevantes.
